# Application of Sulfur and Peroxide Curing Systems for Cross-Linking of Rubber Composites Filled with Calcium Lignosulfonate

**DOI:** 10.3390/polym14091921

**Published:** 2022-05-09

**Authors:** Ján Kruželák, Klaudia Hložeková, Andrea Kvasničáková, Katarína Tomanová, Ivan Hudec

**Affiliations:** Department of Plastics, Rubber and Fibres, Faculty of Chemical and Food Technology, Slovak University of Technology in Bratislava, Radlinského 9, 81237 Bratislava, Slovakia; klaudia.hlozekova@stuba.sk (K.H.); andrea.kvasnicakova@stuba.sk (A.K.); katarina.tomanova@stuba.sk (K.T.); ivan.hudec@stuba.sk (I.H.)

**Keywords:** lignosulfonate, rubber compounds, sulfur curing, peroxide curing, coagent

## Abstract

Calcium lignosulfonate in different loadings was applied to the rubber matrix based on EPDM. A sulfur curing system, organic peroxide, and a combination of organic peroxide with two coagent types were used for cross-linking of rubber compounds. The work was focused on the investigation of filler content and curing system composition in the curing process, cross-link density, morphology, and physical–mechanical properties of composites. The achieved results demonstrated that the curing parameters of rubber compounds cured with the sulfur system were significantly different from those cured with peroxide systems. There was also an observed different influence of curing systems composition on cross link density, though in all cases, the degree of cross-linking showed a decreasing trend with increasing content of lignosulfonate. The tensile strength of the composites cured with sulfur system and organic peroxide was comparable, regardless of lignosulfonate loading. This points to the application of both curing systems in cross-linking of rubber compounds with biopolymer filler. However, the introduction of coagents in peroxide vulcanization led to the improvement of adhesion and compatibility between the rubber and the filler on the filler–rubber interface. This subsequently resulted in the improvement of the tensile characteristics of composites. The introduction of organic peroxide in combination with coagent seems to be a very simple and efficient way for the preparation of biopolymer-filled composites with applicable physical–mechanical properties.

## 1. Introduction

Lignin is a natural polymer, which is one of the three basic wood elements and the second most spread biological material in the world. It is a nontoxic, three-dimensional aromatic polymer that is composed of phenylpropane units bound together by etheric and C-C bonds. Generally, lignin represents an abundant, low-cost, by-product material obtained from the paper and pulp-making industry. From the total amount of lignin isolated from lignin-cellulose materials (≈75 million tons per year), only 1–2% is commercially used. The rest is thrown away or burnt to obtain energy. Despite that, lignin is considered to be a very promising material, which is the source of many chemical substances, but it can also be used as a component in polymer compounds [1,2,3]. The properties and reactivity of lignin are, to a large extent, affected by its origin (coniferous, leafy, one-year plants), composition, heterogeneity of functional groups, and ways of its isolation [4]. Lignin can be extracted from other lignocellulosic parts by chemical, physical, or biochemical treatments. The botanical source extraction procedures and the pulping process (delignification) influence the final structure, purity, and properties [5]. The extraction processes can be classified into two main categories, sulfur, and sulfur-free processes. Sulfur processes are more frequently used, leading to the production of Kraft and lignosulfonate lignins. In the Kraft process, sodium hydroxide and sodium sulfide are used, whereas the sulfite process is acid-catalyzed sulfonating of lignin. It is based on the delignification of wood with aqueous sulfur dioxide and calcium, magnesium, sodium, potassium, or ammonium salt-based acid. Lignosulfonates represent about 88% of all lignin-derived compounds [4]. They contain a higher amount of sulfur and less free phenolic hydroxyl and carboxyl groups in comparison with Kraft lignins. On the other side, sulfonating in an acid environment imparts lignosulfonates ionic exchange, emulsifying, deflocculation, and dispersive properties.

Even though lignin is mostly considered a waste product with minimum commercial importance, a lot of scientific work has been published, unveiling a great application potential of lignin as an additive in polymer compounds. Lignin has been incorporated into polymer matrices to improve thermal [6,7] and thermo-oxidative stability [8,9,10], flame retardancy [11,12], as plasticizer [13], but mostly as a filler [14,15,16,17]. One of the biggest tasks arising from its application into polymers is to achieve good distribution and homogenous dispersion within polymer matrices. Lignin is not a classical nanosized particle-like filler but rather a cross-linked biopolymer filler with a branched structure and different molecular weights. Moreover, because of its hydrophilic character, the compatibility with nonpolar polymer matrices is rather low. Therefore, the introduction of virgin lignin into polymer matrices is usually connected with the deterioration of their physical–mechanical properties [6,14,18,19]. On the other hand, polar functional groups of lignin provide space for its surface modification to improve adhesion and compatibility between the polymer and the filler on the filler–polymer interface. Various chemical and physical methods have been introduced for the modification of lignin to improve its compatibility with polymers and subsequently to improve the physical–mechanical properties of the final materials. Those have been described in many scientific works, for example [1,6,20,21,22,23]. The experimental works clearly demonstrated that the surface modification of lignin is a promising way to fabricate polymer composites with incorporated biopolymer fillers that exhibit useful and applicable properties. Nevertheless, many modifications require experimental procedures that are costly and time-consuming.

The current work deals with the fabrication of composites based on ethylene propylene diene rubber filled with biopolymer filler—calcium lignosulfonate in different concentrations. For cross-linking of rubber compounds, a standard sulfur curing system, organic peroxide, and a combination of organic peroxide with coagents have been applied. The selection of the curing system depends mainly on the type of rubber matrix but also on the preferential characteristics of cross-linked materials. Each curing system leads to the formation of different cross-link spatial network structures within the rubber matrix, which subsequently influence the properties of the final products. Curing systems also have various chemical natures and structures with different functional groups, and thus they also might provide different behavior not only towards rubber matrices but also towards fillers incorporated into polymers.

Sulfur and peroxide curing systems have been most frequently used as cross-linking agents for elastomer matrices in rubber technology. Sulfur vulcanization is the oldest method and still the most widely applied for unsaturated elastomers. During this complex and intricate process, sulfur-based linkages (monosulfidic-, disulfidic-, and polysulfidic-) are formed between rubber chain segments [24]. Sulfur cured vulcanizates exhibit good elastic and dynamic properties, high tensile and tear strength, and good abrasion resistance. On the other hand, they have a low compression set, low thermo-oxidative, and heat aging stability. The sulfur vulcanization process runs in three general stages. In the first stage, the interactions between the curing additives lead to the formation of transition complexes, which, together with rubber, form an active cross-linking agent. In the second stage, a primary vulcanizate network is formed where rubber chain segments are cross-linked mainly with polysulfidic linkages. During the third stage, the primary network is restructured because of the modifications and shortening of polysulfidic cross links and modifications of rubber chains, and a final three-dimensional spatial vulcanizate network is generated [25,26].

Not only unsaturated but also saturated rubbers can be efficiently cured with organic peroxides. Peroxide vulcanization is a radical process during which carbon–carbon linkages are formed within rubber matrices. Organic peroxide first decomposes at curing temperature. Peroxide-derived radicals subsequently react with functional groups of rubber with the formation of radicals on polymer chains. Those radicals usually mutually recombine. Carbon–carbon bonds have higher dissociation energy when compared with sulfur cross-links; thus, the main feature of peroxide cured rubbers are higher resistance to thermo-oxidative aging and higher thermal stability [27,28]. However, when compared with sulfur-cured vulcanizates, they are characterized by lower elasticity and lower tensile characteristics [29]. To improve cross-linking and physical–mechanical properties of the peroxide-cured materials, the so-called coagents are often introduced [30,31,32]. Coagents are low molecular weight organic compounds showing high reactivity towards free radicals. They increase the cross-linking efficiency of peroxide vulcanization, and by homopolymerization and grafting onto rubber chains, they contribute to the improvement of physical–mechanical properties of final rubber articles [33,34]. Moreover, it has been stated that some coagents can also enhance the adhesion toward polar materials [35,36].

EPDM is one of few rubbers that can be efficiently cured with sulfur as well as peroxide vulcanization systems. The chemism and mechanism of both curing processes are different, and thus different types of linkages between rubber chain segments are generated. Not only the type of rubber but also the presence of other components and additives in rubber compounds can influence the reaction pathways during cross-linking. To such components belong mostly fillers, but also antidegradants or other additives. The focus of the work was to investigate the influence of curing systems on the cross-linking process of lignosulfonate-filled rubber compounds and their physical–mechanical properties. By observation of the surface structure, the possible influence of curing systems on the enhancement of adhesion between the biopolymer filler and rubber is discussed.

## 2. Experimental

### 2.1. Materials

Ethylene propylene diene monomer rubber EPDM (KEP 570F, ethylene content—70 wt.%, ethylidene norbornene content—4.5 wt.%) was supplied from Kumho Polychem Co. Ltd., Seoul, Korea. Calcium lignosulfonate under trade name Borrement CA120 provided by Borregaard Deutschland GmbH, Karlsruhe, Germany, was used as biopolymer filler. The elemental analysis of the filler is presented in Table 1. The pH of calcium lignosulfonate was 4.5 (10% solution) with a specific surface area of 3.9 m^2^·g^−1^ and an average molecular weight of 24,000 g·mol^−1^. Calcium lignosulfonate was incorporated into the rubber matrix with a concentration scale ranging from 10 to 60 phr.

In formulation 1, the sulfur curing system consisted of stearic acid and zinc oxide (Slovlak, Košeca, Slovakia) as activators and an accelerator N-cyclohexyl-2-benzothiazole sulfenamide CBS (Duslo, Šaľa, Slovakia) and sulfur (Siarkopol, Tarnobrzeg, Poland) were used for cross-linking of the rubber compounds. Dicumyl peroxide DCP (Sigma-Aldrich, St. Louis, MO, USA) was applied as a peroxide-curing agent either solely (formulation 2) or with a combination of coagents (formulations 3 and 4). Two types of coagents were tested, namely zinc dimethacrylate ZDMA in solid form and trimethylolpropane trimethacrylate TMPTMA in liquid form. Both chemicals were provided by Sigma-Aldrich, St. Louis, MO, USA. The composition of rubber compounds is formulated in Table 2.

### 2.2. Methods

#### 2.2.1. Preparation and Curing of Rubber Compounds

The rubber compounds were compounded in the chamber of a laboratory kneader Brabender (Brabender GmbH & Co. KG, Duisburg, Germany), at 90 °C and 55 rpm. The mixing process was performed in two steps. First, the rubber was plasticated for 1 min, then activators of the sulfur-curing system were added. Calcium lignosulfonate was introduced after 2 min, and the rubber mixture was compounded for the next 4 min. After the first step of mixing, rubber compounds were additionally homogenized and cooled by using the two-roll mill. CBS and sulfur were introduced in the second step, and the mixing process continued in the kneader for 4 min at 90 °C with rotor speed set to 55 rpm. Finally, the rubber compounds were sheeted using the two-roll calender.

The compounding procedure of rubber formulations with peroxide-curing systems proceeded following the same conditions (90 °C, 55 rpm, and an overall mixing time of 10 min). The rubber and the filler were compounded in the first step, which took 6 min, and additives of curing systems were applied in the second step. Final homogenization and sheeting were accomplished using the two-roll mill.

The curing process of rubber compounds was performed at 170 °C and pressure of 15 MPa in a hydraulic press Fontijne (Fontijne, Vlaardingen, Holland) following their optimum cure time. After curing, thin sheets with dimensions 15 × 15 cm and a thickness of 2 mm were obtained.

#### 2.2.2. Determination of Curing Characteristics

Curing characteristics of rubber compounds were determined from corresponding curing isotherms, which were investigated in oscillatory rheometer MDR 2000 (Alpha Technologies, Akron, OH, USA).

The investigated curing parameters were:

∆M (dN.m)—torque difference—the difference between the maximum and minimum torque;

t_c90_ (min)—optimum curing time;

t_s1_ (min)—scorch time;

R_v_ (min^−1^)—curing rate index, defined as:

(1) $$R_{v}=\frac{100}{t_{c90}-t_{s1}}$$

R′ (dN.m·min^−1^)—curing rate, defined as:

(2) $$R^{\prime }=\frac{M_{c90}-{\;M}_{s1}}{t_{c90}-{\;t}_{s1}}$$

M_c90_—torque at t_c90_;

M_s1_—torque at t_s1_

#### 2.2.3. Determination of Cross Link Density

Determination of cross link density *ν* is based on equilibrium swelling of composites in a suitable solvent. Samples of vulcanizates were swelled in xylene within time until the equilibrium swelling was reached. The experiments were carried out at a laboratory temperature, and swelling time was equal to 30 h. The Krause modified Flory–Rehner equation [37] for filled vulcanizates was introduced to calculate the cross link density based upon the previously obtained equilibrium swelling degree.

#### 2.2.4. Investigation of Physical–Mechanical Characteristics

The tensile properties of composites were evaluated using Zwick Roell/Z 2.5 appliance (Zwick Roell Group, Ulm, Germany). The crosshead speed of the measuring device was set to 500 mm·min^−1^, and the tests were carried out in compliance with valid technical standards. Dumbbell-shaped test specimens (thickness 2 mm, length 80 mm, width 6.4 mm) were used for measurements.

#### 2.2.5. Microscopic Analysis

The surface morphology and microstructure of composites were observed using a scanning electron microscope JEOL JSM-7500F (Jeol Ltd., Tokyo, Japan). The samples were first cooled down in liquid nitrogen under glass transition temperature and then fractured into small fragments with a surface area of 3 × 2 mm. The fractured surface was covered with a thin layer of gold and put into the microscope. The source of electrons was a cold cathode UHV field emission gun, the accelerated voltage ranged from 0.1 kV to 30 kV, and the resolution was 1.0 nm at 15 kV and 1.4 nm at 1 kV. SEM images were captured by CCD-Camera EDS (Oxford INCA X-ACT).

## 3. Results and Discussion

### 3.1. Influence of Lignosulfonate and Curing System Composition on Cross-Linking of Rubber Compounds

EPDM is one of few rubbers that can be efficiently cured with sulfur as well as peroxide vulcanization systems. The chemism and mechanism of both curing processes are different, leading to the formation of different types of linkages between rubber chain segments. Not only the type of rubber but also the presence of other components and additives in rubber compounds can influence the reaction pathways during cross-linking. To such components belong mostly fillers, but also antidegradants and other additives. The main aim of the study was to investigate the influence of curing systems on cross-linking and physical–mechanical properties of rubber compounds filled with calcium lignosulfonate.

**Figure 1:**
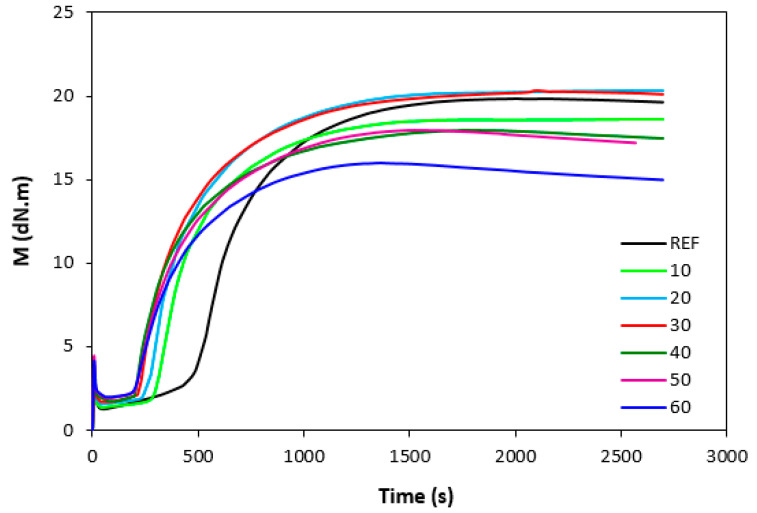
Vulcanization curves of rubber compounds cured sulfur system.

**Figure 2:**
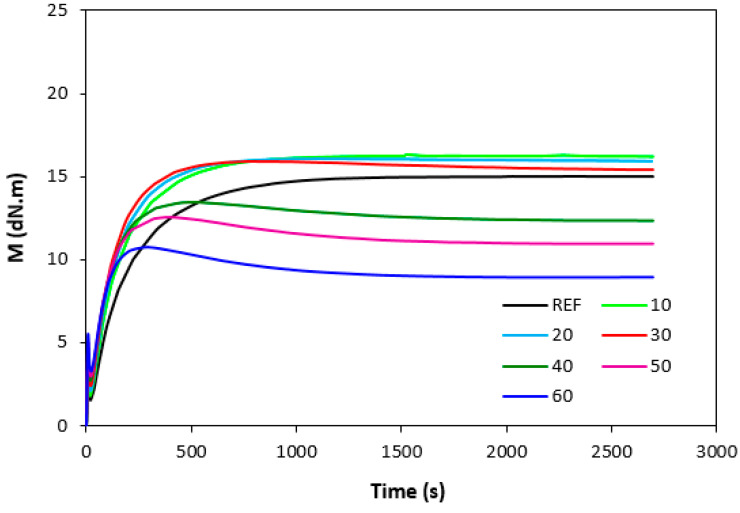
Vulcanization curves of rubber compounds cured with DCP.

**Figure 3:**
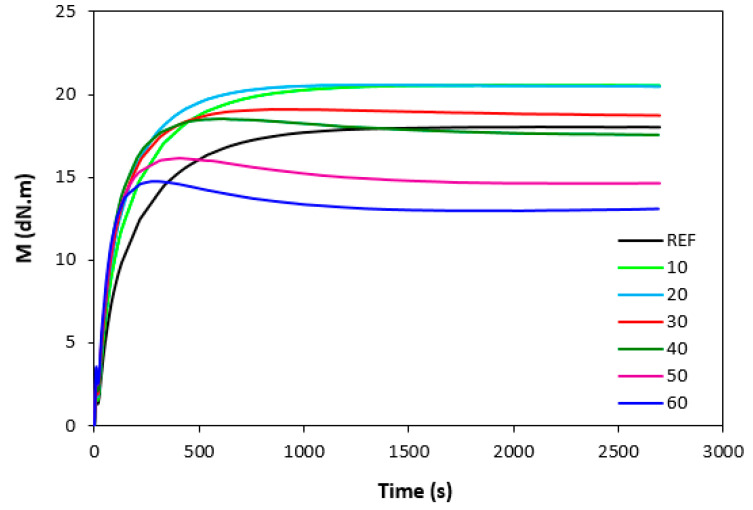
Vulcanization curves of rubber compounds cured with DCP and TMPTMA.

**Figure 4:**
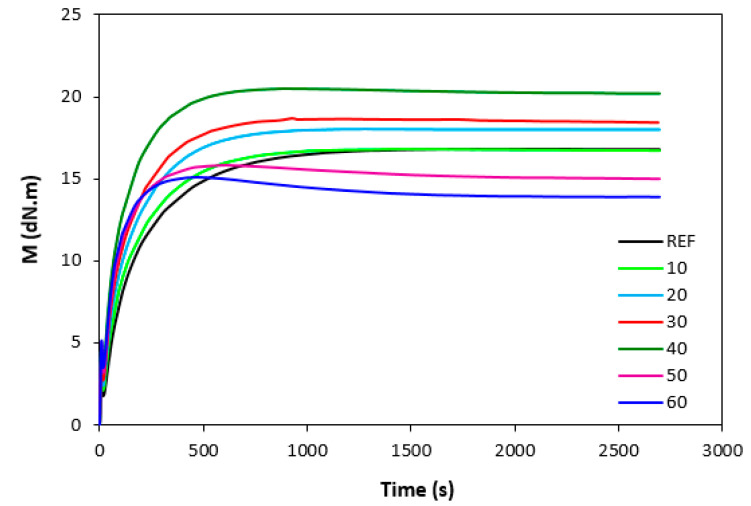
Vulcanization curves of rubber compounds cured with DCP and ZDMA.

**Figure 5:**
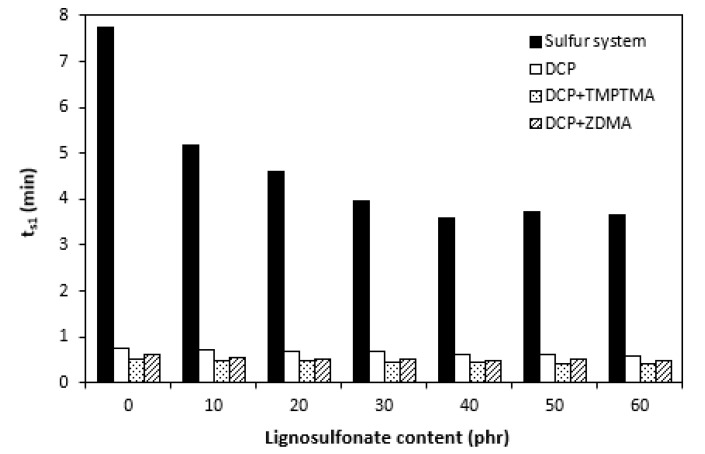
Influence of lignosulfonate content and curing system composition on scorch time t_s1_ of rubber compounds.

**Figure 6:**
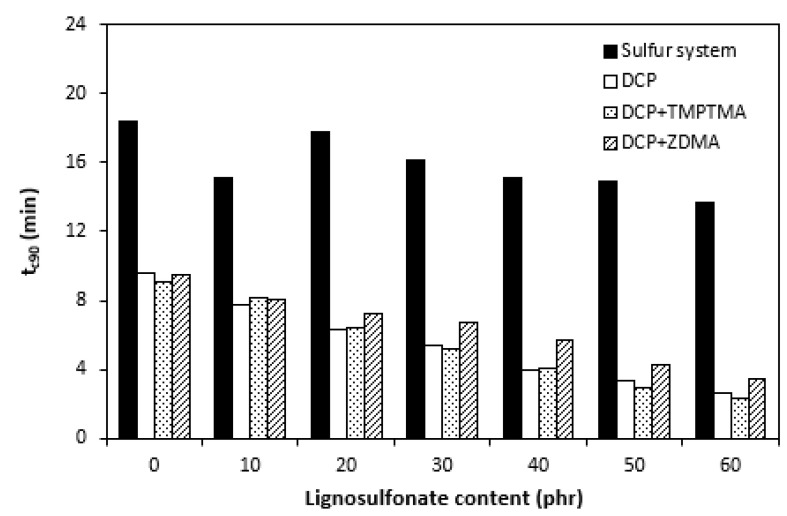
Influence of lignosulfonate content and curing system composition on the optimum cure time t_c90_ of rubber compounds.

The influence of lignosulfonate content and curing system composition on the vulcanization process of rubber compounds was assessed based on the determination of curing characteristics, i.e., optimum cure time t_c90,_ scorch time t_s1_, curing rate R, curing rate index R_v,_ maximum curing rate, and the difference between maximum and minimum torque ΔM. Their values were determined from curing isotherms of corresponding rubber compounds, which are graphically illustrated in Figure 1, Figure 2, Figure 3 and Figure 4. It becomes apparent from them that the type of curing system as well as the content of applied filler influence the vulcanization course. When compared with rubber compounds cured with peroxide systems, sulfur-cured formulations exhibited much higher scorch time and optimum cure time, as also evident from Figure 5 and Figure 6.

**Figure 7:**
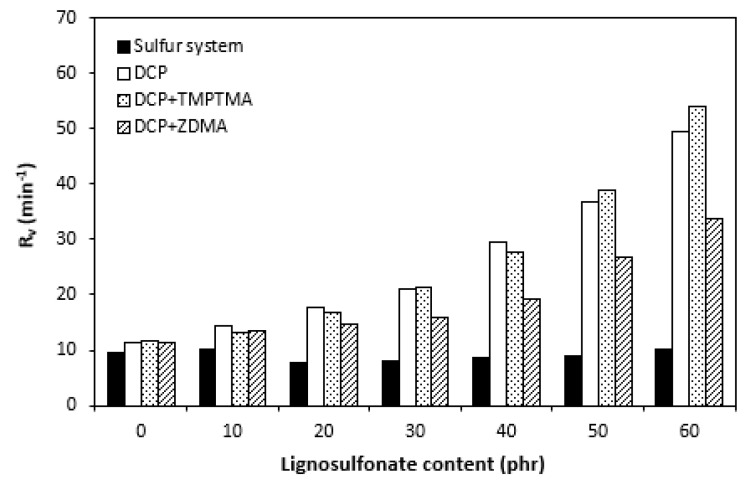
Influence of lignosulfonate content and curing system composition on curing rate index R_v_ of rubber compounds.

**Figure 8:**
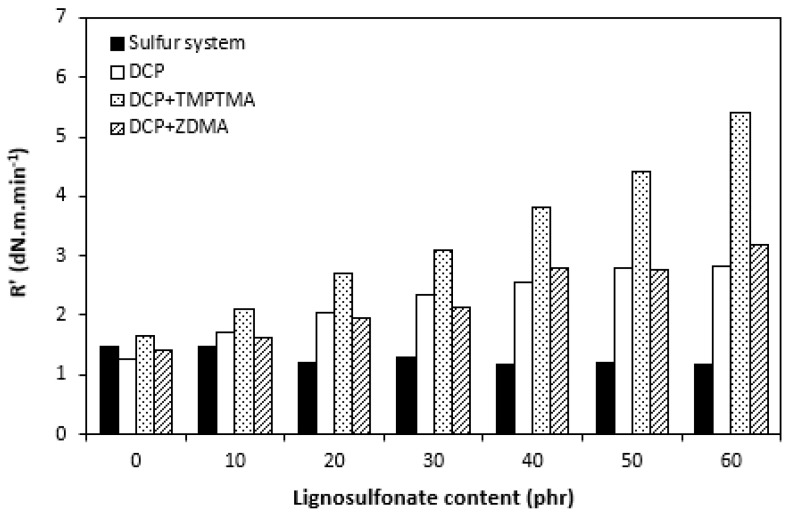
Influence of lignosulfonate content and curing system composition on the curing rate R’ of rubber compounds.

The highest scorch time exhibited reference, unfilled sample. Application of 10 phr of lignosulfonate into rubber compounds cured with sulfur system resulted in a decrease in scorch time in roughly 2.5 min. The scorch time of rubber compounds filled with high lignosulfonate content was more than a half lower when compared with the reference (t_s1_ decreased from almost 8 min for the unfilled sample to 3.5 min for the maximally filled composite). The scorch time of rubber compositions cured with organic peroxide and combination of DCP with coagents was much lower, with almost no influence of the peroxide curing system composition or lignosulfonate content on its values. As seen in Figure 5, the scorch time was only about half a minute regardless of the amount of the filler. Sulfur vulcanization is a very complex process during which the interactions of curing additives take place in the first stage [38,39]. This stage is called the induction period or scorch time. In this period, activators together with accelerators form transition complexes. These complexes react with sulfur, leading to the generation of an active sulfurating agent, which then reacts with functional groups of rubber to form chemical cross links in the main vulcanization course. The length of the induction period, as well as the whole curing process, depends on the composition of the curing system, curing temperature, type of rubber, as well as the presence of other additives in rubber formulations [25]. It is mostly influenced by the type of accelerator. The used accelerator CBS belongs to the delayed action class accelerators that are characterized by a relatively long induction period and fast cross-linking in the main vulcanization course [40]. On the other hand, peroxide vulcanization is a relatively simple process. Organic peroxides first decompose into peroxide active radical species at a vulcanization temperature. Peroxide-derived radicals immediately react with rubber chains leading to the formation of radicals on macromolecular chains. Those radicals usually mutually recombine to form carbon–carbon cross links [41,42]. The length of the induction period can only be hardly controlled by the composition of the peroxide curing system, as it is affected mainly by the type of organic peroxide and its decomposition rate at a vulcanization temperature [35]. The problematic regulation of scorch time is one of the biggest disadvantages of peroxide vulcanization. The reference sample cured with a sulfur system also required the longest time needed for its curing optimum (Figure 6). The application of calcium lignosulfonate resulted in a decrease in optimum cure time. The lowest t_c90_ required the rubber compound filled with maximum lignosulfonate content was almost 14 min when compared with roughly 18 min for the reference. The t_c90_ of rubber compounds with peroxide systems was much lower and showed a decreasing trend with the increasing content of the filler. For closer insight into the kinetics of the curing process, curing rate R’ and curing rate index R_v_ were evaluated. Both parameters serve for a better understanding of the curing process rate, and based on them, it is possible to compare the efficiency of vulcanization systems. It becomes evident from Figure 7 and Figure 8 that the lowest R_v_ and R’ exhibited sulfur cured rubber compositions. Their values seem to be independent of the filler content. As seen, the differences in R_v_ and R’ of the unfilled rubber compounds were negligible, but as the loading of lignosulfonate became higher, the differences in both parameters among rubber compounds cured with different vulcanization systems grew higher, too. In contrast to the curing rate index, the curing rate characterizes not only the time required to achieve the curing optimum but also accounts for the transformation degree of uncured rubber compounds into vulcanizates. Based on the achieved result, it might be stated that the curing process of rubber compounds with a peroxide system proceeded much faster when compared with sulfur-cured equivalents. The incorporation of calcium lignosulfonate resulted in further acceleration of the peroxide curing process.

**Figure 9:**
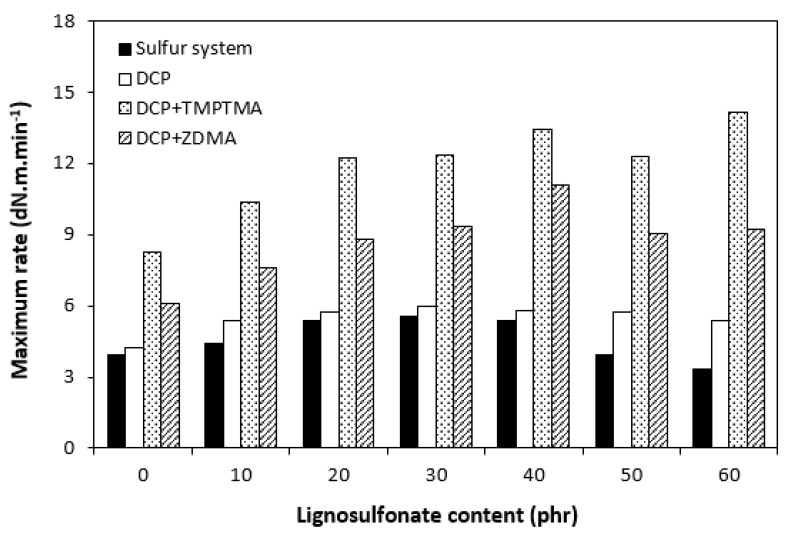
Influence of lignosulfonate content and curing system composition on maximum curing rate of rubber compounds.

Considering the highest R_v_ and mainly R’ parameters of rubber compounds cured with DCP and TMPTMA, mainly at higher filler loading, it might be stated that their curing process proceeded the fastest. That statement can also be confirmed by the highest maximum curing rate of rubber compounds cured with organic peroxide in combination with trimethylolpropane trimethacrylate (Figure 9). On the other hand, although scorch time and optimum cure time of sulfur rubber compounds showed decreasing trend with increasing content of calcium lignosulfonate, the curing rate and curing rate index were found to be independent of the filler loading. The maximum curing rate even decreased at high filler loadings. This suggests that the influence of the filler content on the sulfur-curing process was lower when compared with peroxide-cured equivalents.

**Figure 10:**
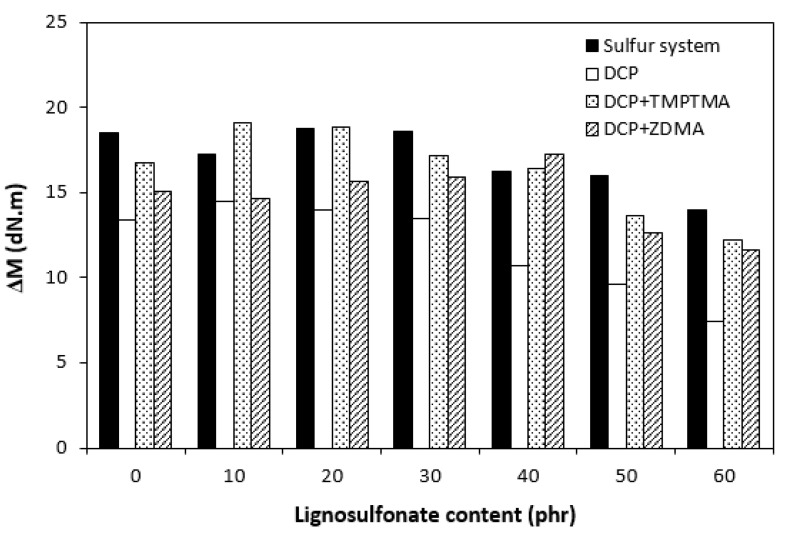
Influence of lignosulfonate content and curing system composition on torque difference ΔM.

**Figure 11:**
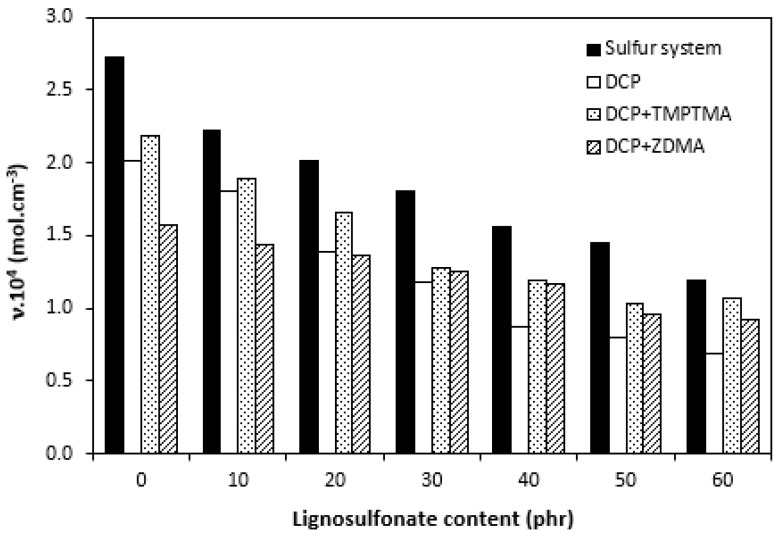
Influence of lignosulfonate content and curing system composition on the cross-link density υ of composites.

There was also a recorded different influence of lignosulfonate content on the difference between the maximum and minimum torque. As shown in Figure 1, Figure 2, Figure 3 and Figure 4, minimum torque slightly increased with increasing content of the filler in sulfur as well as peroxide curing processes, which can be attributed to the increasing viscosity of rubber compounds because of the presence of the filler. With the exception of the formulations filled with 20 phr and 30 phr of the filler, the maximum torque of sulfur-cured rubber compounds filled with higher lignosulfonate content was lower when compared with the reference. This was also reflected in decreasing trend of torque difference ΔM for highly filled rubber compounds (Figure 10). The increment of maximum torque was also observed for DCP-cured rubber compounds filled with lower filler content (up to 30 phr) (Figure 2). Then, the maximum torque of highly filled rubber compounds dropped below the corresponding value of the reference. A similar situation was recorded for rubber compounds cured with organic peroxide in combination with coagents (Figure 3 and Figure 4). The lowest maximum torque of rubber compounds cured with DCP was also the reason for the lowest torque difference ΔM of the equivalent rubber compounds. Looking at Figure 10, it can be stated that the differences between the maximum and minimum torque of rubber compounds changed only slightly at lower filler content. Then, the decrease in ΔM values was observed for rubber compounds with high lignosulfonate content. When comparing Figure 10 and Figure 11, one can also see a certain correlation between torque difference and cross link density. As rubber compounds cured with DCP showed the lowest torque difference in curing isotherms, they also exhibited the lowest cross-link density. On the other hand, the highest cross link density was found to have composites cured with a sulfur system with a high torque difference. The decreasing trend of torque difference (mainly for highly filled rubber compounds) suggests the decreasing trend of cross-linking degree with increasing content of lignosulfonate, which was clearly confirmed by experimental determination of cross link density (Figure 11).

As shown, the highest cross link density exhibited reference vulcanizate cured with a sulfur system. Then, the increasing degree of filling resulted in a decrease in cross-link density. Taking into consideration that calcium lignosulonate contains sulfur in its structure, the decrease in the cross link density seems to be a bit surprising. The possible explanation could be the fact that part vulcanization system might preferentially react with sulfur in the filler structure leading to the cross-linking of the filler itself rather than cross-linking of the rubber matrix. In this case, the efficient concentration of curing system additives might be reduced and insufficient for cross-link formation between rubber chains. Another explanation might be the fact that sulfur in the filler structure is chemically bound and does not participate in cross-linking or react with activators and accelerators to form inactive species. Other theories are based on the assumption that calcium lignosulfonate could act as a steric hindrance against cross link formation within the rubber matrix or that the acidic character of lignosulfonate might decelerate the cross-linking process. It is generally known that acidic substances have negative effects on the vulcanization course [40,43]. The cross-linking degree of rubber compounds cured with peroxide systems was slightly lower in comparison with sulfur-cured equivalents and similarly showed decreasing trend with increasing content of the filler. As peroxide curing of rubber runs on a different mechanism, the role of sulfur in the filler structure is not under consideration. Three possible explanations can be applied. First, as in the previous case, it is based on filler itself acting as a steric hindrance against cross-link formation. The second might be postulated on literature knowledge claiming that peroxide-derived radicals can preferentially react with substances containing easily abstractable hydrogen atoms, mainly those containing hydroxyl groups. The negative effect of some antidegradants (mainly phenolic types) on the peroxide vulcanization of rubbers has been reported [44]. Lignosulfonates contain hydroxyl groups that might behave in a similar way. The reactive sites could be formed on the filler preferentially reacting with other filler particles leading to the cross-linking of lignosulfonate. The third explanation is also supported by general findings revealing that acidic substances present during the vulcanization process can cause heterolytic or ionic decomposition of peroxides [45,46]. As a result, no radicals are formed, so cross-linking does not take place. Detailed analysis of lignosulfonate demonstrated its acidic character (pH = 4.5). However, this theory is not supported by the acceleration of the peroxide-curing process with increasing content of the filler. Another possible theory explaining the decrease in cross link density for both sulfur- and peroxide-cured vulcanizates might be the polarity of lignosulfonate because it could absorb polar curing reagents and make them ineffective during vulcanization [47]. Also, sometimes fillers cause decomposition of the curing additives in a faster way to produce faster crosslinking, but because of the nonrecyclable catalytic activity, they can produce a lower number of cross links [48]. To fully understand to role of different curing systems in cross-linking of rubber compounds filled with lignosulfonate, deeper insights and other experiments will be necessary to perform.

### 3.2. Influence of Lignosulfonate and Curing System Composition on Properties and Morphology of Composites

**Figure 12:**
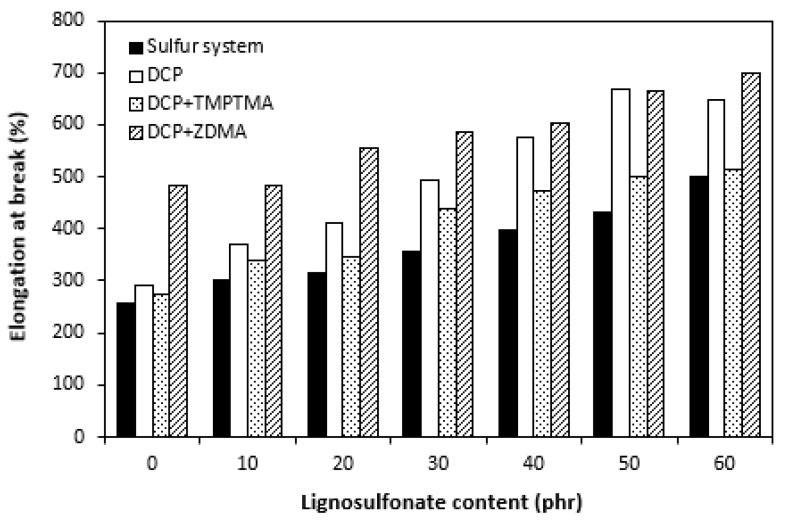
Influence of lignosulfonate content and curing system composition on elongation at break of composites.

**Figure 13:**
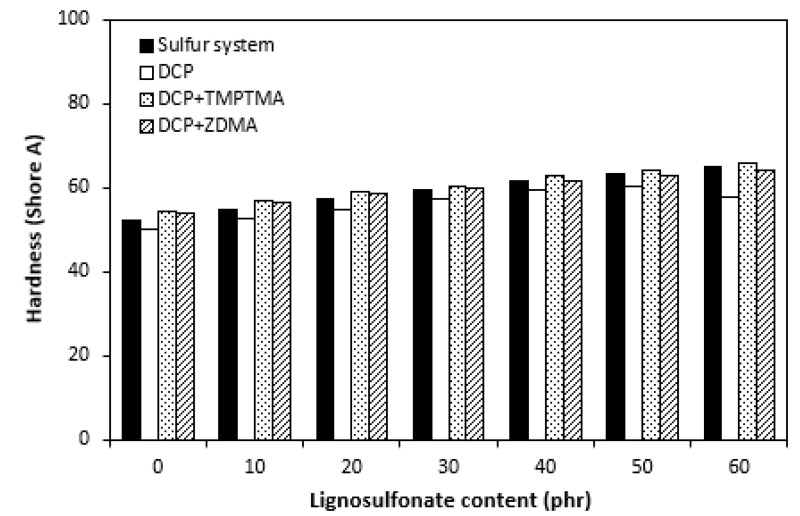
Influence of lignosulfonate content and curing system composition on the hardness of composites.

The dependences of elongation at the break on curing system composition and content of lignosulfonate were found to be in close correlation with the cross link density. When comparing Figure 11 and Figure 12, one can see that composites cured with a sulfur system with the highest cross link density exhibited the lowest elongation at the break. The higher the cross link density, the lower the mobility and elasticity of rubber chain segments, and thus the elongation at break decreases. On the other hand, composite materials cured with organic peroxide and a combination of DCP and ZDMA showing the lowest cross-linking degree were found to have the highest elongation at the break. Also, the decreasing trend of cross-link density with increasing lignosulfonate content resulted in the increase in elongation at break for all composites. The highest elongation at break exhibited composites filled with maximum lignosulfonate content. The influence of the curing system composition or filler content on hardness very low, though it could be said that the higher was the amount of calcium lignosulfonate, the higher was the hardness of composites (Figure 13).

**Figure 14:**
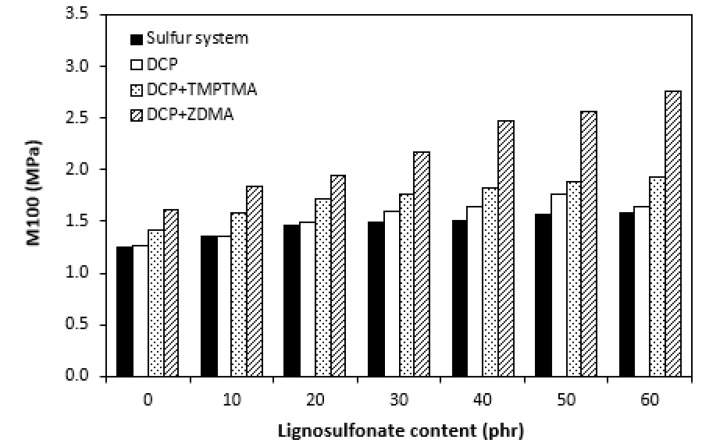
Influence of lignosulfonate content and curing system composition on modulus M100 of composites.

**Figure 15:**
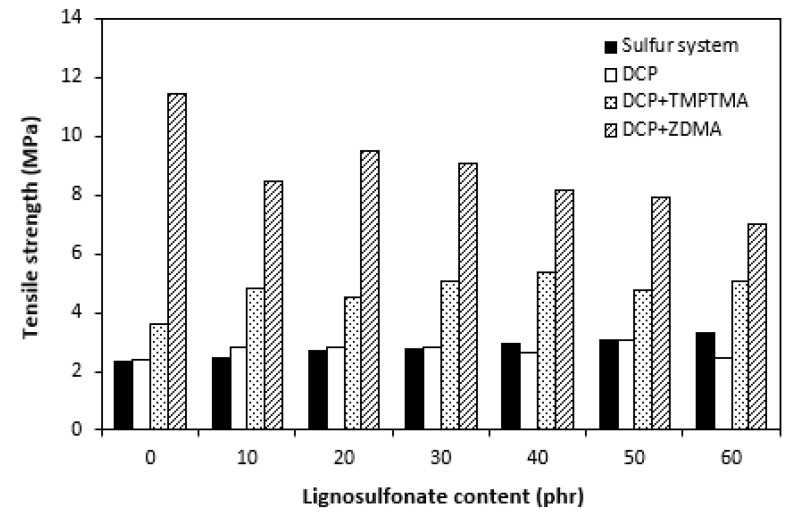
Influence of lignosulfonate content and curing system composition on tensile strength of composites.

As calcium lignosulfonate is not a typical particulate filler but rather a polymer-based filler, its influence on hardness was not significant. Little influence of lignosulfonate content on hardness could also be caused by decreasing trend of cross-link density, depending on filler content. As shown in Figure 14, composites cured with a sulfur system demonstrated the lowest modulus M100 in the whole filler concentration range. This might be a bit surprising as, usually, moduli are also in close correlation with cross link density. In general, the higher the cross-link density, the higher the moduli. The modulus of composites cured with the sulfur system, organic peroxide, and combination of organic peroxide with trimethylolpropane trimethacrylate were very similar, with low dependence on lignosulfonate content. The highest M100 and its highest dependence on filler content demonstrated composites cured with dicumyl peroxide and zinc dimethacrylate. The differences in modulus among composites were more pronounced with increasing content of lignosulfonate. From Figure 15, it becomes apparent that there was almost no difference in tensile strength of composites cured with sulfur system and organic peroxide, not even in dependence on lignosulfonate content. The fact that unmodified lignins as well as lignosulfonates mostly behave as inactive fillers when incorporated in polymer matrices and deteriorate their physical–mechanical properties, no negative influence of tested lignosulfonate on composite properties seems to be very promising. It suggests the high application potential of EPDM-based rubber composites filled with calcium lignosulfonate even at high filler loadings. Second, as there was almost no difference in physical–mechanical properties of composites cured with sulfur system and dicumyl peroxide, it can be stated that rubber compounds based on EPDM filled with lignosulfonate can be efficiently cured with both sulfur systems and organic peroxides. As both curing systems have certain advantages and disadvantages, the selection of vulcanization system can be properly considered on the basis of the final product application and the requirement for their useful properties. As also seen in Figure 15, the introduction of coagents in peroxide-curing systems resulted in the improvement of tensile strength. The tensile characteristics of composites cured with DCP and TMPTMA were about one and a half or two times higher in comparison with equivalent composites cured either with the sulfur system or organic peroxide. The application of calcium lignosulfonate resulted in an improvement in tensile strength of the composites cured with DCP and TMPTA (from about 3.5 MPa for the reference to above 5 MPa for the composite with maximum lignosulfonate content). Even if this is not a very significant improvement, it is another promising factor suggesting the application potential of lignosulfonate-filled rubber composites. The highest tensile strength was found to have the reference sample cured with DCP and ZDMA (almost 11.5 MPa, which is significantly higher when compared with reference vulcanizates cured with other systems). Although the application of lignosulfonate resulted in a slight deterioration of tensile characteristics, they still provided the highest tensile strength among all tested composites.

The tensile strength of cured rubber compounds is a complex property, which is influenced not only by the cross link density but also by the structure of the formed physical and chemical cross links within the rubber matrix, the type of rubber, and the type and content of the filler, too. In the case of filled rubber systems, the mutual compatibility and adhesion between the rubber and filler on the filler–rubber interface are very important aspects influencing the tensile behavior of composites. As outlined, coagents contribute to the formation of a complex cross link structure within the rubber matrix by homopolymerization and grafting onto rubber chains. On the other hand, as they are polar molecules, they are supposed to improve adhesion to polar materials, which can be ranked as lignosulfonates as well [49,50,51,52]. By chemical coupling onto rubber chains and physical adhesion to lignosulfonate, coagents can improve the compatibility and homogeneity of the filler–rubber interface. The overall reinforcement of the rubber matrix can be thus achieved, which subsequently resulted in the improvement of physical–mechanical characteristics of composites, mainly in the enhancement of tensile strength. In addition to the ability of ZDMA to form chemical and physical cross links within the rubber matrix and to improve adhesion to lignosulfonate, a higher tensile strength of composites cured with DCP and ZDMA can be attributed to the formation of ionic cross links or ionic clusters through static electronic attractions with the contribution of zinc ions from coagent molecules. Composite systems with this type of morphology demonstrate the ability of stress relaxation that is due to rubber chain slippage on the surface of ionic clusters and recovery of ionic bonds upon the influence of external strains [53,54,55].

**Figure 16:**
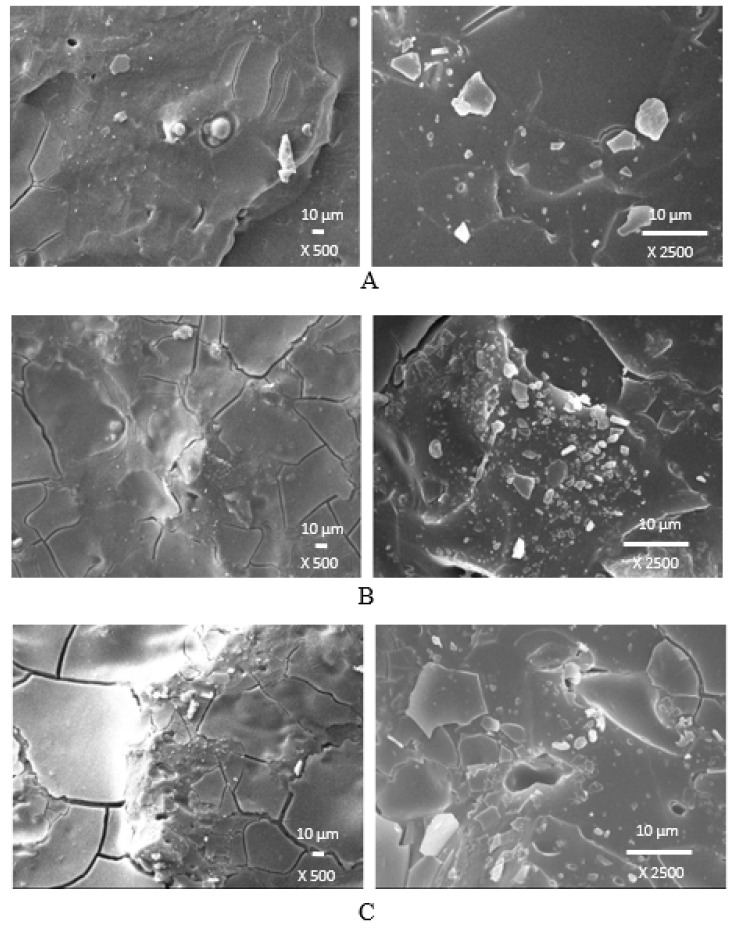
SEM images of composites cured with a sulfur system containing 10 phr of lignosulfonate (**A**), 30 phr of lignosulfonate (**B**) a 60 phr of lignosulfonate (**C**).

**Figure 17:**
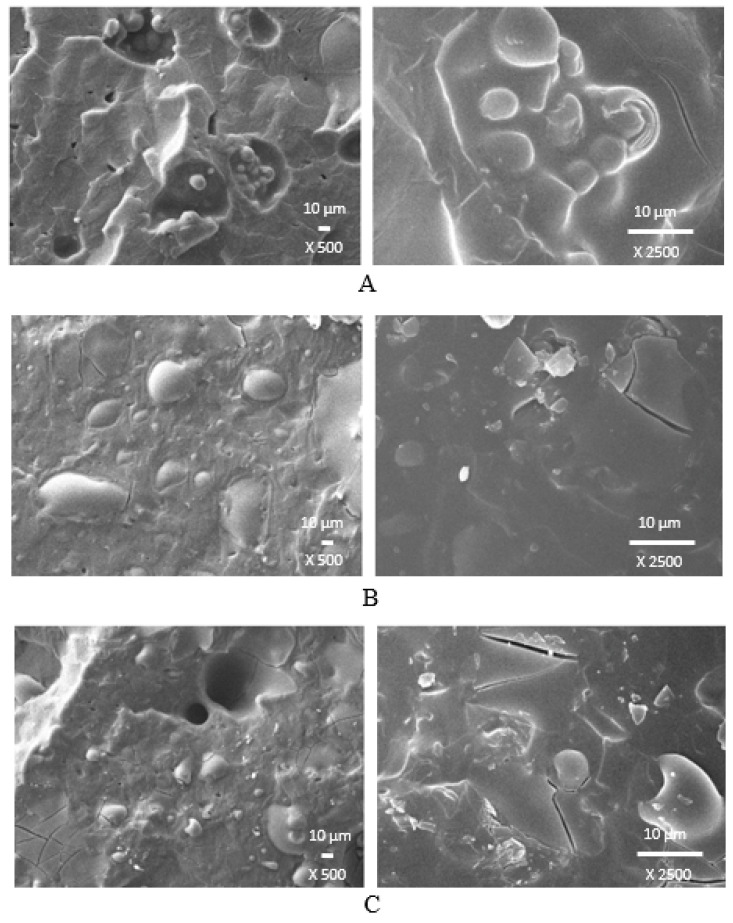
SEM images of composites cured with dicumyl peroxide and trimethylolpropane trimethacrylate containing 10 phr of lignosulfonate (**A**), 30 phr of lignosulfonate (**B**), and a 60 phr of lignosulfonate (**C**).

Microscopic images revealing the microstructure and morphology of surface fractures of composites cured with a sulfur system and a combination of DCP with TMPTMA are presented in Figure 16 and Figure 17. From SEM images of composites cured with a sulfur system (Figure 16), it is shown that lignosulfonate forms lower or bigger agglomerates, which could be caused by insufficient dispersion of the filler within the rubber matrix. It could also be deduced that the mutual compatibility and homogeneity between the rubber matrix and the filler are not very good. The reason might be attributed to the poor adhesion between both components, which was probably due to incompatibility in polarity (polar filler dispersed in nonpolar rubber matrix). On the other hand, surface fractures of composites cured with DCP and TMPTMA suggest that mutual adhesion between the rubber and the filler is higher, which can be attributed to the presence of a coagent (Figure 17). As already outlined, coagents might increase the polarity of the rubber matrix because of chemical bonding onto rubber chains, and by physical interactions with lignosulfonate, they could contribute to the improvement of adhesion between the rubber and the filler on their interface.

## 4. Conclusions

Calcium lignosulfonate-filled rubber compounds based on EPDM were cured with sulfur and peroxide systems. The main aim was to investigate the influence of curing system composition and lignosulfonate content on cross-linking, morphology, and physical–mechanical properties of composites.

The results revealed that the curing process of rubber compounds with a sulfur system takes significantly longer. Based on the determination of curing kinetics parameters, it can be stated that lignosulfonate has an accelerating effect mainly on the vulcanization process of rubber compounds cured with peroxide systems. The application of lignosulfonate into rubber compounds resulted in the decrease in cross link density of composites cured with sulfur as well as peroxide systems. As a result, the elongation at break showed an increasing trend with an increase in filler loading. The highest cross-linking degree of composites cured with a sulfur system was reflected in the lowest elongation at the break of the equivalent composites. The influence of filler content and curing system composition on hardness was less visible. The compatibility between the rubber and the filler in composites cured with the sulfur system was low, while the application of coagents in the peroxide system is supposed to improve the adhesion and homogeneity of the filler–rubber interface. This was subsequently reflected in the enhancement of modulus and mainly tensile strength of composites.

## Figures and Tables

**Table 1:** Elemental analysis and the number of hydroxyl groups (-OH) in calcium lignosulfonate.

Composition	N	C	H	S	-OH
wt.%	0.14	46.63	5.35	5.62	1.56

**Table 2:** Composition of rubber compounds with sulfur and peroxide curing systems.

	EPDM	ZnO	Stearic Acid	CBS	Sulfur	DCP	TMPTMA	ZDMA	Filler
Form. 1	100	3	2	1.5	1.5				0–60
Form. 2	100					1			0–60
Form. 3	100					1	10		0–60
Form. 4	100					1		10	0–60

## Data Availability

Not applicable.
